# New horizons in gynecological surgery: first-year experience with HUGO™ robotic-assisted surgery system at two tertiary referral robotic centers

**DOI:** 10.1007/s13304-024-01902-7

**Published:** 2024-06-10

**Authors:** Margarita Afonina, Claudia Collà Ruvolo, Giorgia Gaia, Marco Paciotti, Giovanni Leva, Anna Maria Marconi, Koen Traen, Alexandre Mottrie

**Affiliations:** 1 Department of Obstetrics and Gynecology, San Paolo Hospital Medical School, University of Milan, Milan, Italy; 2 Department of Obstetrics and Gynecology, Onze-Lieve-Vrouwziekenhuis, Aalst, Belgium; 3 ORSI Academy, Ghent, Belgium; 4 Department of Urology, Onze-Lieve-Vrouwziekenhuis, Aalst, Belgium; 5 Department of Neurosciences, Reproductive Sciences and Odontostomatology, School of Medicine, University of Naples “Federico II”, Naples, Italy; 6 Department of Urology, Humanitas Research Hospital, IRCCS, Rozzano, Italy

**Keywords:** Robotic surgery, HUGO RAS system, Gynecological procedures, Minimally invasive surgery

## Abstract

The HUGO™ robotic-assisted surgery system (RAS, Medtronic, CA) consists of a 3D open console, four independent carts, and an integrated laparoscopic and robotic tower. Approved in 2021, it represents a novel alternative platform for robotic procedures. The aim of our study is to report the first-year experience with this system for gynecological procedures at two tertiary referral robotic centers. We prospectively collected and retrospectively analyzed data from patients underwent gynecological robot-assisted surgery with the HUGO™ RAS, at San Paolo University Hospital (Milan, Italy), and Onze Lieve Vrouw (OLV) Hospital (Aalst, Belgium), March 2022–April 2023. Demographic characteristics, intraoperative settings, and perioperative outcomes were investigated. A total of 32 procedures were performed: 20 (62.5%) hysterectomies, 7 (21.9%) adnexal surgeries, and 5 (15.6%) pelvic floor reconstructive surgeries. In 2022 and 2023, 13 (40.6%) and 19 (59.4%) procedures were carried out, respectively. The median docking time was 8 min (IQR 5.8–11.5). The median console and skin-to-skin time was 52.5 min (IQR 33.8–94.2) and 108.5 min (IQR 81.5–157.2), respectively. No intraoperative complications occurred. Two conversions to laparoscopy managed without any additional complications were needed. To the best of our knowledge, this is the first global series of gynecological procedures performed with the HUGO™ RAS. Our preliminary findings showed the system’s feasibility reporting promising results. The observed upward trend in the total number of procedures during the analyzed period is encouraging. Further studies are needed to assess a standardized method in the gynecological field with the novel platform.

## Introduction

Nowadays, robotic surgery represents a solid treatment option in the gynecological field thanks to its amplified 3D visualization, precision, and freedom of movement. Depicting the cornerstone of robotics, the Intuitive Da Vinci® surgical system has been the only platform available until the late 2010s when some of its patents expired. Since then, new competitors have joined the scene keeping the advantages of this surgery, while cutting costs and making the robotic approach mostly accessible. In this regard, the HUGO™ Robot-Assisted Surgery (RAS) System (Medtronic, Minneapolis, MN, USA) received conformité européenne mark approval in 2021 and was promptly adopted at our tertiary referral centers (San Paolo Hospital Medical School, University of Milan, Milan, Italy and Onze-Lieve-Vrouwziekenhuis, Aalst, Belgium). Thereafter, several studies have been conducted for urological and general surgery procedures, underlining the feasibility and versatility of the novel platform [1–8]. Conversely, only one case series and three case reports are published in the scientific literature regarding the gynecological field [9–12]. The current study aimed to report our first-year experience with the HUGO™ RAS system for both benign and malignant gynecological indications at two tertiary referral multiplatform robotic centers.

## Materials and methods

### Study population

We collected data from consecutive women who underwent robotic surgery between March 2022 and April 2023 at San Paolo University Hospital (Milan, Italy), and Onze-Lieve-Vrouwziekenhuis Hospital (Aalst, Belgium). All the procedures were carried out with the novel HUGO™ RAS system by two robotic expert surgeons, after a dedicated training consisting in an e-learning followed by the wet lab practice, at the ORSI Academy, Melle, Belgium.

### Variable definitions

The following characteristics were recorded for each patient: age (continuously coded), body mass index (BMI, kg/m^2^), Charlson Comorbidity Index (CCI), previous abdominal surgery (yes or no), and the surgical indication (endometrial hyperplasia, leiomyomas, benign ovarian tumors, endometriosis, pelvic organ prolapse, or endometrial cancer).

The surgical procedures were classified as follow: hysterectomy, adnexal surgery (defined as salpingectomy, ovarian cystectomy or adnexectomy), and pelvic floor surgery (defined as promontofixation or lateral suspension according to Dubuisson technique) ± supracervical hysterectomy.

For each procedure, docking, console and skin-to-skin times (minutes) were registered. In addition, clashing of the instruments, technical errors or system failures, conversion rate, estimated blood loss (cc), perioperative complications defined with the Clavien–Dindo classification, day of catheter removal, and length of stay (LOS, days) were reported.

### The HUGO™ RAS system, operative setting, and port placement

The HUGO™ RAS system consists of a 3D open console, four fully independent carts, which need to be docked one by one, and an integrated laparoscopic and robotic tower. For all the cases, a 4 arms configuration, according to the Medtronic compact set up guide, was used. Before port placement and docking process all arm-carts were parked at 45–60 cm from the operative table, two by each side. First, the endoscope 11 mm port was placed on the midline, 1.5–2 cm above the umbilicus. Left 8 mm robotic port was placed under vision at least 14 cm on the same transversal line of the endoscopic port. To the right side, two 8 mm robotic ports were placed, the medial one, at least 8 cm far, and 5 cm below from the endoscopic port and the lateral one, at least 14 cm far, and on the same transversal line of the endoscopic port. Finally, the assistant 12 mm port carrying the airseal® insufflation system was placed in the left or right hypocondrium according to the surgeon preferences. Monopolar Curved Shears, Bipolar Maryland Forceps, Needle driver and Fenestrated Grasper or Cadiere Forceps were used for all surgeries.

The patient was placed in Lloyd Davies 20° Trendelenburg position, with the legs opened and supported by Allen® stirrups to allow uterine manipulation. ClearView® uterine manipulator was used for the adnexal surgeries, Hohl (Storz®) manipulator was used for the benign hysterectomies, Shar (Storz®) for the pelvic floor procedures and no manipulation was used for the malignant indications. The pneumoperitoneum was kept at 8 mmHg during all surgeries. For pelvic floor and malignant indications, a 30° endoscope was used, in all the other procedures a 0° camera was adopted.

### Statistic analysis

Descriptive statistics were presented as medians with the interquartile ranges (IQR) for continuously coded variables or counts and percentages for categorically coded variables. A LOESS curves was used to depict the docking time from the first (March 2022) to the last (April 2023) procedure. Box and whiskers plots were used to depict console and skin-to-skin time according to surgical procedure. In all statistical analyses, the R software (www.rproject.org) environment for statistical computing and graphics (R version 4.0.0) was used.

## Results

During the observation period a total of 32 procedures were carried out: 13 (40.6%) in 2022 and 19 (59.4%) in 2023. Of all, 15 (46.9%) and 17 (53.1%) surgeries were performed at the San Paolo Hospital and at the and Onze-Lieve-Vrouwziekenhuis Hospital, respectively (Table 1).

**Table 1:** Baseline characteristics of 32 patients who underwent gynecological robot-assisted procedures with HUGO RAS system at Onze Lieve Vrouw Hospital (Aalst, Belgium) and San Paolo University Hospital (Milan, Italy), from November 2022 to April 2023

		Overall (*n* = 32)
Age	Median	51.5
	IQR	40.8–61.8
BMI (kg/m^2^)	Median	24.3
	IQR	21.4–27.1
CCI	0	13 (40.6)
	1	8 (25.0)
	2	5 (15.6)
	3	6 (18.8)
Previous abodminal surgery	Yes	14 (43.8)
	No	18 (56.2)
Surgical indication	Endometrial hyperplasia	9 (28.1)
	Leiomyomas	6 (18.8)
	Benign ovarian tumors	5 (15.6)
	Endometriosis	5 (15.6)
	Pelvic organ prolapse	5 (15.6)
	Endometrial cancer	2 (6.2)
Surgical procedure	Hysterectomy	20 (62.5)
	Adnexal surgery	7 (21.9)
	Pelvic floor surgery + / − supracervical hysterectomy	5 (15.6)
Year of surgery	2022	13 (40.6)
	2023	19 (59.4)
Center	San Paolo	15 (46.9)
	OLV	17 (53.1)

*BMI* Body mass index, *CCI* Charlson Comorbidity Index, *IQR* interquartile range, *OLV* Onze Lieve Vrouw

### Baseline characteristics

The overall median age was 51.5 years (IQR 40.8–61.8). The overall median BMI was 24.3 kg/m^2^ (IQR 21.4–27.1). The CCI was 0, 1, 2, and 3 for 13 (40.6%), 8 (25%), 5 (15.6%), and 6 (18.6%) patients, respectively. A history of previous abdominal surgery was recorded in 14 (43.8%) patients. Two (6.2%) patients were treated due to endometrial cancer and 30 (93.8%) patients underwent surgery for benign indications (endometrial hyperplasia [*n* = 9], leiomyomas [*n* = 6], benign ovarian tumors [*n* = 5], endometriosis [*n* = 5], and pelvic organ prolapse [*n* = 5]). Hysterectomy was the most performed procedure (*n* = 20, 62.5%), followed by adnexal surgery (*n* = 7, 21.9%), and pelvic floor surgery ± supracervical hysterectomy (*n* = 5, 15.6%, Table 1).

### Perioperative characteristics and follow-up

**Fig. 1:**
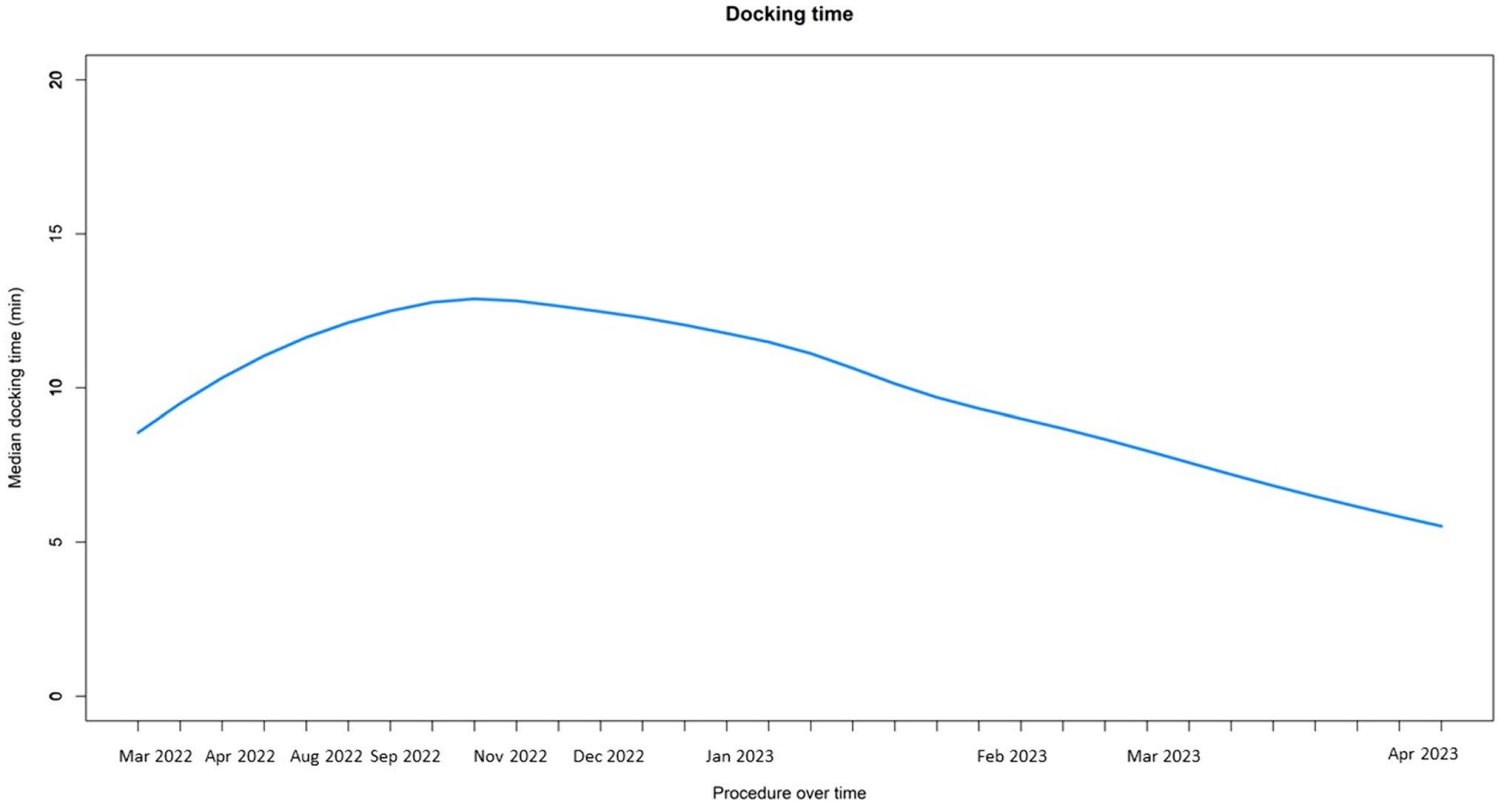
Graphical representation of the docking time of 32 robot-assisted surgical procedures performed with HUGO RAS system from March 2022 to April 2023 (LOESS)

**Fig. 2:**
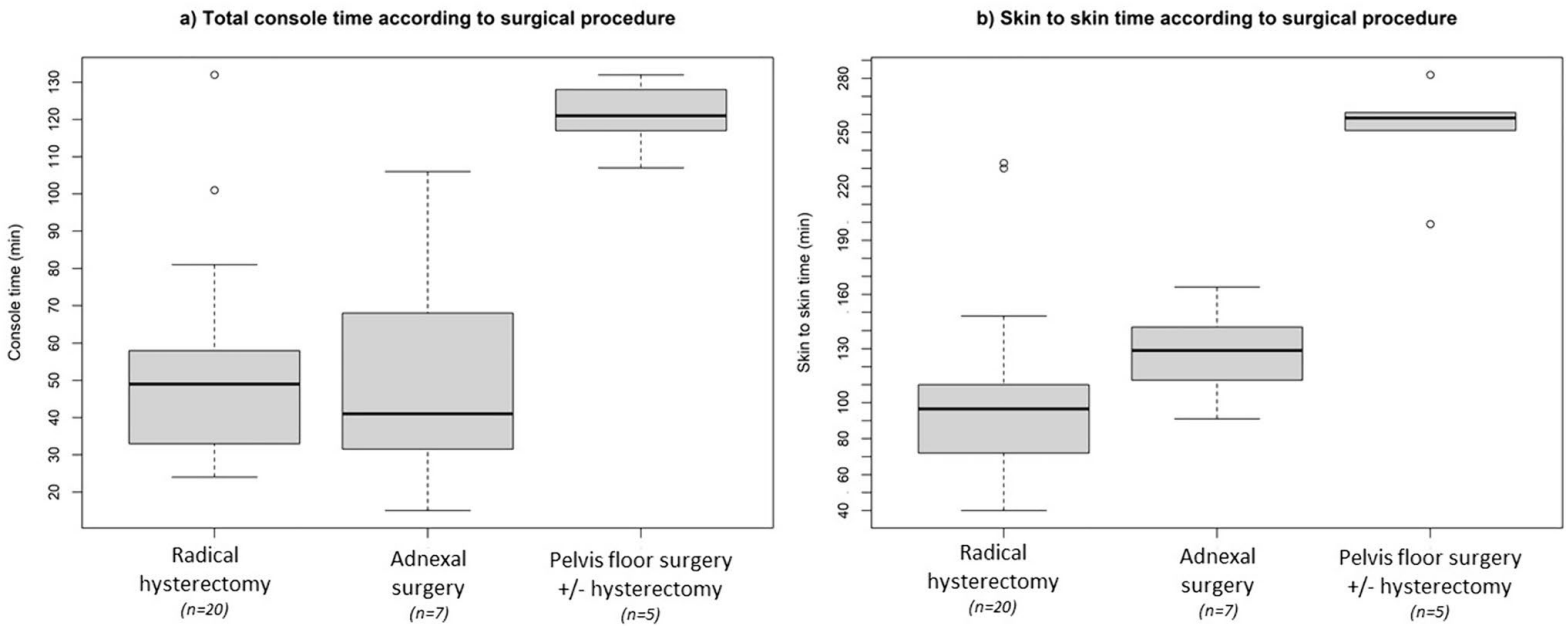
Box and whiskers plots depicting console time (**a**) and skin-to-skin time (**b**) according to surgical procedure. Boxes denote the interquartile range. The solid black horizontal bar denotes the median. Whiskers denote the 95% range of the distribution of console and skin-to-skin time. The open circles denote outlier values

The median docking time was 8 min (IQR 5.8–11.5) and ranged from 11 to 8 min from the first to the last procedure (Fig. 1). The median console time was 52.5 min (IQR 33.8–94.2) and was 49 min (IQR 33.5–57) for radical hysterectomy, 41 min (IQR 31.5–68) for adnexal surgery and 121 min (IQR 117–128) for pelvic floor procedures ± supracervical hysterectomy (Fig. 2a). The median skin-to-skin time was 108.5 min (IQR 81.5–157.2) and was 96.5 min (IQR 72.5–110) for radical hysterectomy, 129 min (IQR 112.5–142) for adnexal surgery and 258 min (IQR 251–261) for pelvic floor procedures ± supracervical hysterectomy (Fig. 2b) (see Table 2).

**Table 2:** Intra and post operative characteristics of 32 patients who underwent gynecological robot-assisted procedures with HUGO RAS system at Onze Lieve Vrouw Hospital (Aalst, Belgium) and San Paolo University Hospital (Milan, Italy), from November 2022 to April 2023

		Overall (*n* = 32)
Docking time (min)	Median	8
	IQR	5.8–11.5
Console time (min)	Median	52.5
	IQR	33.8–94.2
Skin-to-skin time (min)	Median	108.5
	IQR	81.5–157.2
Blood loss	Median	20
	IQR	8.2–100
Length of stay (days)	1	17 (53.1)
	2	11 (34.4)
	3	4 (12.5)
Catether removal (post-operative day)	1	31 (96.9)
	2	1 (3.1)
Clavien–Dindo classification	No	30 (93.8)
	I	0 (0)
	II	0 (0)
	III	1 (3.1)
	IV	1 (3.1)
	V	0 (0)
Conversion	No	30 (93.8)
	Yes	2 (6.2)

IQR interquartile range

The median blood loss was 20 cc (IQR 8.2–100). One (3.1%) patient was admitted to the intensive care unit after hysterectomy for an acute hypercapnia, completely recovered the day after (Clavien–Dindo grade IV). One (3.1%) patient was readmitted 1 week after hysterectomy, presenting unilateral hydronephrosis, treated by ureteral reimplantation (Clavien–Dindo grade III). Two (6.3%) conversions to laparoscopy due to console malfunction were recorded. Both procedures were managed laparoscopically without any additional complications. Median LOS was 1 day (IQR 1–2), and the urinary catheter was removed on postoperative day one for all the women (IQR 1–1). Here is the right place for the citation of table 2. (see Table 2). 

### Postoperative management

The same postoperative management was applied to all cases. Intravenous fluids were administered until the patient was able to eat and drink normally. Continuous saline irrigation was performed for the first hours, and then slowed and stopped as the urine clears. In all standard cases, the catheter was removed on postoperative day one.

## Discussion

In this study, we reported the surgical outcomes of the gynecological procedures performed with the HUGO RAS system after 1 year of its use at two tertiary referral multiplatform robotic centers. Overall, 32 procedures for benign and malignant indications were carried out, more than half in the last 4 months of the observation period (March 2022–April 2023), indicating the feasibility of the new platform in this field.

Since the approval of robotic surgery for the gynecological procedures by the Food and Drug Administration in 2005, the number of procedures has been increasing year by year. Nevertheless, there is still no agreement and no established guidelines for choosing robotics over traditional laparoscopy. In addition, the costs associated with robotic surgery limit its widespread adoption [13]. In this scenario, the recent introduction of new robotic contenders in the global market plays a pivotal role. Among the new available robotic platforms, the HUGO™ RAS system has already been reported to be safe and feasible, especially for urological procedures [1, 3]. However, mostly case reports and only one case series, which focused exclusively on pelvic floor reconstructive surgery, are currently available regarding the application of HUGO™ RAS in the gynecological field [9–12].

In this manuscript, we reported the pioneering experience of two of the first centers that have adopted this new platform in their gynecological departments. From the current study, several key considerations can be highlighted.

The trocar placement and the arm carts docking for the HUGO™ RAS platform is different from the established Da Vinci® system. Specific gynecological setup guides are provided by the Medtronic company leading to an easier initiation to the new system. However, thanks to the modular setup, surgeons maintain a certain level of flexibility to find their own optimal configuration. We reported a median docking time of 8 min, without showing a relevant change from the first (11 min) to the last surgery (8 min). Our data are concordant to Mottaran et al. study which describes the first five robotic sacropexy [14] performed with HUGO™ RAS system at Onze-Lieve-Vrouwziekenhuis Hospital (Aalst, Belgium) reporting a median docking time of 8 min. However, these data must be interpreted in the context of centers that already had extensive robotic experience, and future studies conducted in robotic-naive centers are necessary to better understand the learning curve process.

Regardless of the initial approach to the new platform, more than 90% of the procedures were successfully completed. Moreover, we have described a wide range of procedures including adnexal surgeries, simple hysterectomies, and more complex cases, such as pelvic floor reconstructive surgery or deep infiltrating endometriosis influencing only the procedure time, without compromising the surgery successful rate. Similar outcomes were reported by Panico et al. Specifically, the authors described 60 sacrocolpopexies, and in most of the surgery (93.3%) a subtotal hysterectomy, a total hysterectomy, or a salpingo-oophorectomy were uneventfully performed, proving the system versatility. It must be reported that in our experience two laparoscopic conversions were necessary due to a system malfunction. Both procedures were effectively managed laparoscopically, without encountering any other complications, thanks to the expertise gained from previous laparoscopic background at our centers. Although unpleasant complications, these events have confirmed an easy switch to a laparoscopic approach thanks to the integrated tower of the HUGO™ RAS system. It is important to report that, after the latest system updates, no platform failures were recorded anymore. This finding is consistent with the experiences of other groups [4, 15].

Regardless of the center’s expertise, the training of the entire (and possibly multiple) operating room team plays a crucial role when approaching robotic surgery. This remains true when transitioning from one system to another or when multiple platforms are available at the same center. The training should be mandatory for the entire team, starting from the console surgeon, extending to the bedside assistants, including the nurses. In our experience, two different centers, with two different backgrounds, equips, and different expertise were involved, bringing to remarkable results. Both surgical teams received the same training at ORSI Academy, Melle, Belgium, starting from technical aspects of the system (setup of docking and tilt arm carts angle, emergency undocking in case of emergency scenario or mechanical instrument release in case of systems malfunctions) to procedural training on a live porcine model.

Finally, one of the most debated factors influencing whether to opt for robotics in gynecology and the system choice is the economic aspect. A recent comprehensive analysis of the available literature on emerging robotic surgical platforms in urology revealed limited information on cost-effectiveness, making it challenging to establish if these platforms are less expensive compared to the traditional Da Vinci® system [16]. However, despite the absence of specific data, it is noteworthy that as competency and familiarity with a robotic platform increase, leading to improvement in the surgical technique, a steep learning curve, and optimizing the robotic approach, the associated costs generally diminish. On the other hand, opening the market to new platforms may allow robotic surgery approach even to smaller centers extending the availability of high precision minimally invasive surgery.

There are two major limitations in this study that could be addressed in future research. First, a small sample size is reported. However, to the best of our knowledge, this is the largest worldwide series of several gynecological procedures for benign or malignant indication performed with the novel HUGO™ RAS system. However, we reported data from two multiplatform robotic centers with the availability to perform surgery with several systems, without a favor selection, which may partially explain the small sample size. Second, all the surgeons involved are experts in robotic surgery. This should be considered when interpreting the obtained results. Despite this, all the surgeons had limited experience with HUGO™ RAS system and were therefore still on their learning curve for this platform. In this context, working in a high-volume robotic center, equipped with various robotic systems and access to different robotic platforms and simulators, confers a distinct advantage in the learning curve [17].

## Conclusion

To the best of our knowledge, this is the first global series of several gynecological procedures performed with the HUGO™ RAS. Our initial findings showed the platform’s feasibility reporting promising results from two centers characterized by different equips and various expertise. The uneventful conversion to laparoscopy, facilitated by the integrated tower, highlights the technology’s remarkable versatility. In addition, the observed upward trend in the total number of procedures performed during the analyzed period is encouraging. Further studies are needed to assess a standardized method in the gynecological field with the new platform.

## Data Availability

Raw data were generated at San Paolo University Hospital, Milan, Italy, and OLV Hospital , Aalst, Belgium. Derived data supporting the findings of this study are available from the corresponding author M. A. on request.
